# A Case Report on the Incidental Diagnosis of a Left Atrial Myxoma in a Patient Presenting With Right Shoulder Pain and Inter-scapular Back Pain

**DOI:** 10.7759/cureus.23187

**Published:** 2022-03-15

**Authors:** Zahid Khan, Yousif Yousif, Mohammed Abumedian, Mildred Ibekwe, Vinod Warrier, Syed Aun Muhammad, Animesh Gupta

**Affiliations:** 1 Cardiology, Royal Free Hospital, London, GBR; 2 Internal Medicine, Barking, Havering and Redbridge University Hospitals NHS Trust, London, GBR; 3 Geriatrics, Barking, Havering and Redbridge University Hospitals NHS Trust, London, GBR; 4 Internal Medicine, Mid and South Essex NHS Foundation Trust, Southend on Sea, GBR; 5 Cardiology, Mid and South Essex NHS Foundation Trust, Southend on Sea, GBR; 6 Acute Internal Medicine, Barking, Havering and Redbridge University Hospitals NHS Trust, London, GBR

**Keywords:** transthoracic echocardiogram, inspiratory dyspnea, coronary ct angiogram, cardiac troponin, atypical chest pain, non valvular atrial fibrillation, asymptomatic myxoma, left atrial mass

## Abstract

Primary cardiac tumors are rare, and myxoma is a rare benign primary cardiac tumor in adults, commonly found within the left atrium. The presentation can vary from patients being asymptomatic to pulmonary embolism or stroke. Smaller atrial myxomas are usually asymptomatic, however, larger ones can cause symptoms such as dyspnea, orthopnea, cough, peripheral edema, palpitations, and fatigue. We present a case report of a 72-year-old patient presenting with right shoulder pain and chest pain on breathing to the accident and emergency department. The patient was complaining of right shoulder pain for five days and pleuritic chest pain for the last 48 hours. Initial electrocardiogram showed normal sinus rhythm, however, repeat electrocardiograms showed atrial fibrillation. An echocardiogram showed a homogeneous, relatively round mass seen in the left atrium, close to the inter-atrial septum, and close to the roof of the left atrium, and the patient underwent surgical removal of the benign tumor.

## Introduction

Cardiac myxoma is a benign adult cardiac tumor accounting for over 50% of all cardiac tumors with an incidence rate of 0.5% per million per year [1]. The echocardiographic features of atrial myxoma were first described by Effert and Domanig in 1959 and since then, a remarkable increase in the incidence of the tumor has been observed [2].

Primary cardiac tumors are rare, with an estimated incidence of less than 0.03%; of these, 75% are benign and myxomas account for half of them. Myxomas are mostly found in the left atrium (LA) close to the fossa ovalis (FO), however, they are also found in other locations such as the right atrium, ventricles, and valves [3]. The presentation can vary depending on the location of the myxoma such as shortness of breath and chest pain due to mitral valve (MV) obstruction, thromboembolism such as cerebral emboli or pulmonary embolism, or arrhythmia such as atrial fibrillation (AF) [4].

Atrial myxomas (AM), in general, present as 1-15 cm masses ranging from 15 to 180 g and grow at a rate of 1.3-6.9 mm/month [5-6]. Smaller tumors are generally more likely to cause thromboembolism due to villous appearance and friability, whereas larger tumors tend to have a smooth surface and are hence less likely to cause thromboembolism [5]. The reported incidence of systemic embolization in atrial myxoma manifesting as a neurological deficit is around 20-35% [7]. In comparison, AM was found in one out of 250 young adults and one out of 750 older patients who had a transient ischaemic attack (TIA) or stroke [7]. It is important to get the correct diagnosis of atrial myxoma due to significant clinical variation in its presentation.

## Case presentation

A 72-year-old patient presented to the Accident and Emergency (A&E) department with right shoulder pain for the last seven days and stabbing back pain between the shoulder blades on breathing for the last four days. Her past medical history was significant for previous smoking and chronic obstructive pulmonary disease (COPD). Her medications include salbutamol and beclomethasone inhaler only. She denied any fever, cough, or shortness of breath (SOB). She denied any travel history and had no coronavirus disease 2019 (COVID-19) symptoms and both her COVID-19 polymerase chain reaction (PCR) and lateral flow were negative. Her physical examination was unremarkable, the chest was clear on auscultation, and heart sounds were normal.

Her laboratory test results are shown in Table 1.

**Table 1:** Lab test results of the patient on days 1 and 7

Blood test	Normal value	Day 1	Day 7
Haemoglobin	133–173 g/L	136	135
White cell count	(4.0-11.0) 109/L	7.2	5.9
Neutrophil	(1.7-7.5) 109/L	6.3	6.2
C reactive protein (CRP)	0-5 mg/L	49	25
Troponin T	<14 ng/L	19	23
D-Dimer	250–400 ng/ml	1200	750
Sodium	133–146 mmol/L	142	139
Potassium	3.5–5.3 mmol/L	4.9	4.2
Urea	2.5–7.8 mmol/L	6.3	6.5
Creatinine	59–104 umol/L	67	60
Pro brain or B-type natriuretic peptide	<125 pg/mL	1249	560

**Figure 1:**
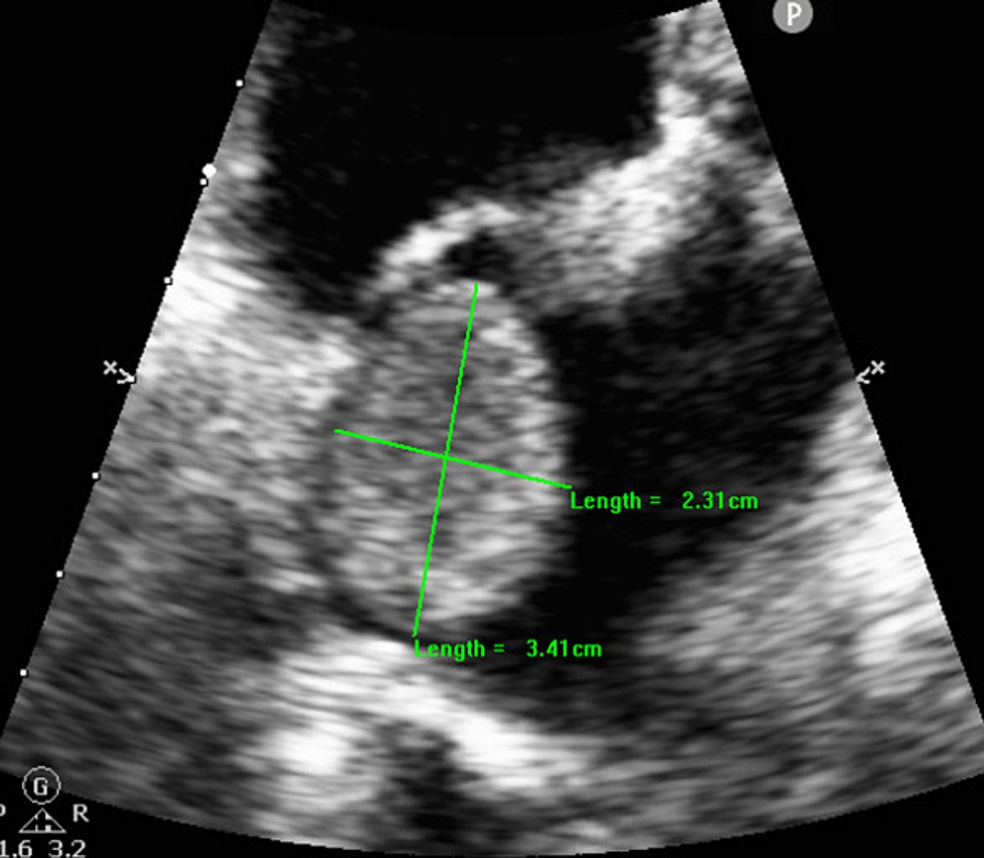
Left atrial myxoma

Her electrocardiogram (ECG) showed normal sinus rhythm (NSR) initially and repeat ECGs showed AF. She had a computerized tomography pulmonary angiogram (CTPA) in view of raised D-dimer and stabbing back pain. Her blood pressure was normal, and her initial heart rate was 75 beats per minute (bpm); however, her heart rate a few hours later jumped up to 150 bpm when the patient developed AF with a rapid ventricular response (RVR). CTPA did not show any pulmonary embolism but showed a mass in the left atria suggestive of an intracardiac tumor. The patient had a bedside echocardiogram showing a large, left-side mass with good left ventricular ejection fraction > 60%. Departmental echocardiogram showed a homogeneous, relatively round mass seen in the left atrium (LA), close to the inter-atrial septum (IAS) and to the roof of LA, and the mass did not seem to protrude toward the left ventricle (LV) through the mitral valve (MV), hence no significant flow obstruction was noted across the MV (mean pressure gradient (MPG) 1.39 mmHg). The findings were suggestive of atrial myxoma. The LV cavity was small in size with overall dynamic systolic function and estimated LVEF > 65% as shown in Figure 1 and Videos 1-3.

**Video 1:** Left atrial myxoma on echocardiogram on parasternal long-axis view (PLAX)

**Video 2:** Left atrial myxoma on echocardiography in apical two chambers view

**Video 3:** Left atrial myxoma on echocardiography in apical two chambers view

She was commenced on Tinzaparin 7000 units in view of new-onset atrial fibrillation and atrial myxoma and was also commenced on bisoprolol 2.5 mg once daily (OD) to control her rate. Her thyroid function tests (TFTs) were normal.

**Figure 2:**
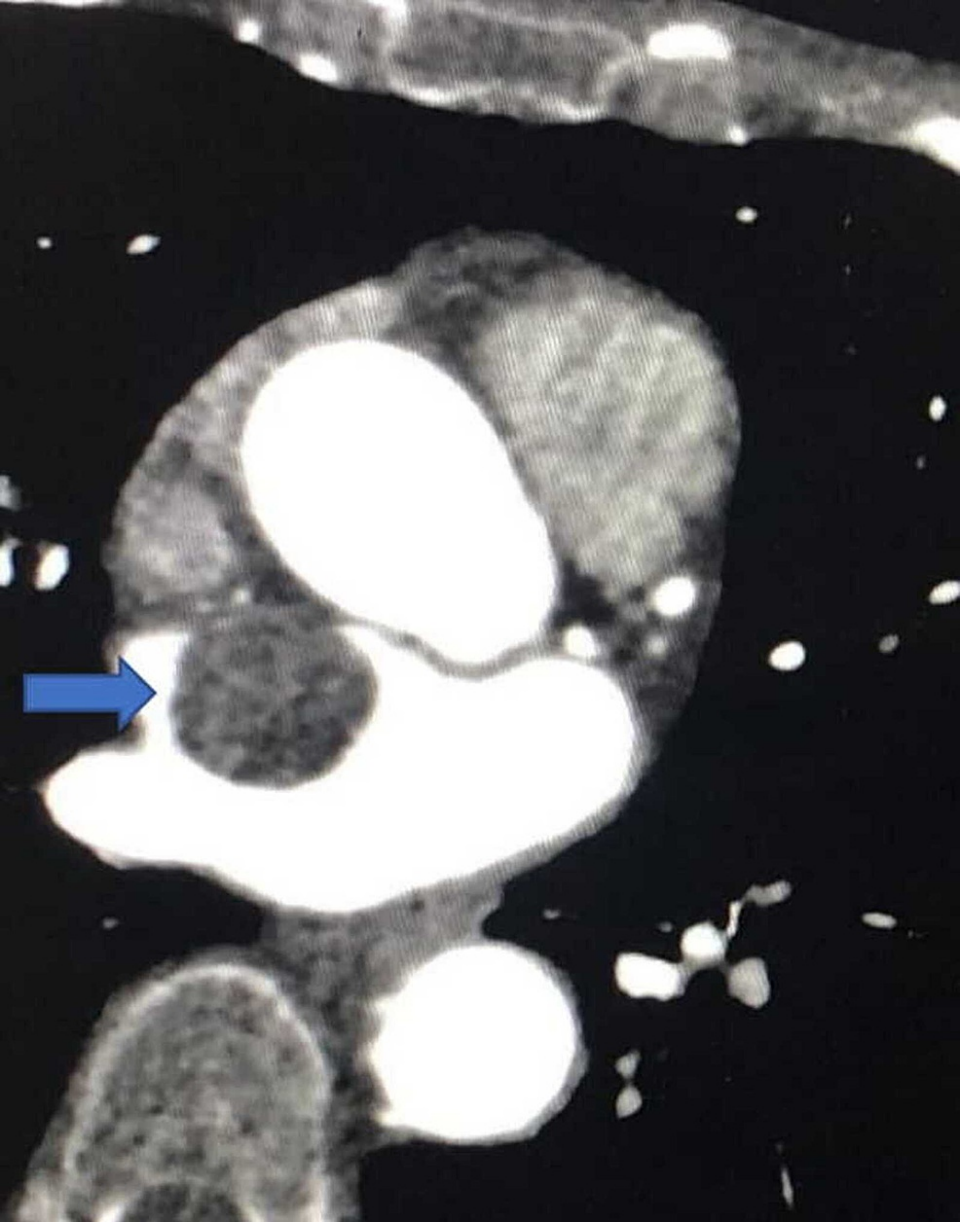
Left atrial myxoma on computerized tomography coronary angiogram

The patient also had computerized tomography coronary angiogram (CTCA) that showed mild coronary artery disease (CAD) (25-49%). The aortic root measured 35 X 33 X 33 mm, ascending thoracic aorta 33 mm, and descending thoracic aorta 29 mm. There was mild atheroma in the thoracic aorta. The CTCA also showed extensive background emphysematous change and mild bilateral bronchial wall thickening and subsegmental atelectasis in the lower lobes and middle lobe. There was a large 34 x 25 x 24 mm low attenuation mass in the left atrium attached to the interatrial septum with a broad base. There is no definitive stalk or calcification, and the left atrial myxoma is at the top of the differential diagnosis as shown in Figure 2.

The patient also had computerized tomography abdomen and pelvis that showed bilobar biliary duct dilatation and dilated CBD with abrupt caliber change at the level of the ampulla. The pancreatic duct diameter was at the upper limit of normal. Left upper pole renal lesion and renal protocol CT scan was advised.

She underwent successful removal of the atrial myxoma and has remained symptom-free since. Histopathological findings were consistent with atrial myxoma and showed loose myxoid stroma with scattered round cells having dense irregular nuclei and inflammatory cells with hemosiderin deposits. She remains in NSR and was commenced on apixaban 5 mg twice daily (BD) on discharge and remains on bisoprolol.

## Discussion

Myxomas are the most common, primary, cardiac benign tumors of the heart. Myxoma is commonly located in the left atrium and mainly originates from an area in the atrial septum near the fossa ovalis. Approximately 90% of cardiac tumors are benign and 75-80% of these are atrial myxomas and 75% of atrial myxomas occur in the left atrium and the remaining 25% in the right atrium [8]. Sometimes. it can be challenging to differentiate a myxoma from a thrombus or malignant tumor on an echocardiogram only. Transoesophageal echocardiography (TOE) can be useful in these patients and one key differentiating feature between a thrombus and myxoma is that the thrombus does not have a stalk. Most intra-atrial septal tumors (IAS), both benign and malignant, are found during percutaneous closure of the patent foramen ovale [9].

The clinical manifestations of an atrial myxoma can vary greatly depending on the location, size, and mobility of the mass, and patients may present with tetrad of symptoms such as arrhythmias, intracardiac flow obstruction, embolic phenomena, and constitutional symptoms [10]. An atrial myxoma is also associated with the Carney complex, and several case reports have been published confirming this association [11].

Atrial myxomas are associated with embolic events such as stroke or transient ischaemic attack (TIA) in about 40-50% of patients [10]. In addition, AM is also associated with embolization to other organs, such as kidneys, spleen, aortic bifurcation, and the lower extremities, and case reports of patients having a stroke and splenic and renal embolization have been reported previously [10,12]. Left atrial myxoma may mimic other valvular abnormalities, such as mitral regurgitation, pulmonary embolism, tricuspid stenosis, and tricuspid regurgitation, and about 36% of patients do not have any murmur on auscultation despite having underlying valvular pathology. A mitral stenosis murmur has been reported in only 54% of patients with atrial myxoma [13-14].

Arterial embolization can also occur in patients with atrial myxoma, and a case report discussed a 48-year-old patient who presented with fever and paraesthesia and underwent bilateral femoral and left brachial embolectomy secondary to a left atrial myxoma [15]. Few case reports of hemolytic anemia and thrombocytopenia associated with left atrial myxomas and mitral stenosis have been reported and the likely explanation for this is the mechanical destruction of these blood elements by a mobile intraluminal tumor although the exact pathogenesis remains unknown [15].

Myocardial infarction (MI) secondary to AM is extremely rare, and the reported incidence is about 0.06%. The likely explanation for this lower incidence of MI is the fact that the coronary apertures form a right-angled junction within the aortic root, thus providing protection to the coronary arteries through the aortic valve cusps [16]. Another study reported a total of 16 myocardial infarction cases secondary to AM between 2003 and 2013 [16]. The incidence of acute myocardial infarction (AMI) was equal in males and females despite the greater frequency of myxomas in women and the patients’ age ranged between nine and 64 years. The most commonly affected wall in patients with AMI was the inferior wall, which was affected in 53% of the cases, and 10 cases out of 17 had a normal coronary angiogram. Most patients with a normal coronary angiogram were younger patients aged < 45 years [16].

Another study reported the association between hypertrophic obstructive cardiomyopathy (HOCM) and AM, and a systemic review performed by them showed six cases from 1970 to 2019 [17]. Two patients in this systemic review did not have any family history of AM but HOCM. Cardiac magnetic resonance imaging (CMR) has been found to be superior in the detection of cardiac tumors [17].

## Conclusions

In conclusion, cardiac tumors are mostly benign, and atrial myxomas are the most common tumors. AM presentation may vary, ranging from embolic phenomenon to acute myocardial infarction, fever, valvular regurgitation, heart failure, syncopal episodes, arrhythmia, and pulmonary embolism. An echocardiogram is useful in the detection of these cases, and CMR is the gold standard to diagnose cardiac tumors. Our patient presented with atypical chest pain and right arm pain and had chest pain likely due to mitral valve obstruction secondary to AM. It is therefore important to be aware of the varied presentation of AM, and it should be considered in patients with atypical presentation and atrial fibrillation.
